# Evaluation of Reproducibility and Accuracy of Facial Soft Tissue Landmarks in Individuals Assessed Using Various 3D Face Scanning Modalities: A Systematic Review

**DOI:** 10.7759/cureus.107230

**Published:** 2026-04-17

**Authors:** Sejal Naikwadi, Suryakant Powar, Wasundhara Bhad, Sumeet Ghonmode, Amal BJ, Sharvari Kadhare

**Affiliations:** 1 Orthodontics and Dentofacial Orthopedics, Government Dental College and Hospital, Mumbai, IND

**Keywords:** 3d facial scanning, accuracy, facial soft tissue landmarks, reproducibility, stereophotogrammetry

## Abstract

The present systematic review evaluated the accuracy and reproducibility of facial soft tissue landmark assessment across different three-dimensional face scanning modalities used in dentofacial practice. The review was conducted in accordance with PRISMA guidelines and registered in PROSPERO (CRD42025628750). Electronic searches of PubMed, Scopus, Web of Science, Cochrane Library, Semantic Scholar, and Google Scholar identified studies assessing facial soft tissue landmarks using 3D facial scanning systems and reporting accuracy and/or reproducibility outcomes. Eighteen cross-sectional studies were included in the qualitative synthesis, comprising 498 participants. The included modalities spanned photogrammetry, laser scanning, stereophotogrammetry, structured-light scanning, and smartphone-based depth/photogrammetry workflows. Comparator methods included direct anthropometry, manual measurements, electromagnetic digitizers, geometric references, 2D photographs, stereophotogrammetry reference systems, and cone beam computed tomography-derived soft tissue models. Accuracy was evaluated in 13 studies, while reproducibility or reliability was reported in all 18 studies. Most studies reported clinically acceptable landmark accuracy and repeatability under standardized conditions, with stronger and more consistent performance generally seen with stereophotogrammetry and structured-light systems. Smartphone-based systems also showed promising and often clinically acceptable results, although performance was more variable across facial regions, particularly in lateral landmarks. Reproducibility was typically better for well-defined midline landmarks than for less distinct lateral soft tissue points. Owing to substantial heterogeneity in scanner types, landmark protocols, comparator standards, and outcome metrics, meta-analysis was not performed and a narrative synthesis was undertaken. Overall, 3D facial scanning appears suitable for soft tissue landmark assessment, but careful device selection and standardized protocols remain essential.

## Introduction and background

Three-dimensional (3D) facial imaging has become an important part of contemporary dentofacial diagnosis, treatment planning, and outcome assessment [1]. In orthodontics and related dental specialties, evaluation of facial soft tissue landmarks is central to understanding facial proportions, symmetry, growth changes, and treatment response [2]. Compared with conventional two-dimensional photographs and manual anthropometry, 3D facial scanning offers the advantage of capturing facial form in all spatial planes, with the potential for more comprehensive and repeatable measurements [3]. As a result, these systems are increasingly being used in clinical practice, academic settings, and research [4].

A wide range of 3D facial scanning modalities is currently available, including laser scanning, structured-light scanning, photogrammetry, stereophotogrammetry, and more recently, smartphone-based depth-sensing systems [3]. These technologies differ in hardware design, acquisition speed, image reconstruction methods, cost, operator dependence, and clinical practicality [5]. While advanced systems such as stereophotogrammetry platforms are often considered reference standards for facial soft tissue imaging, newer portable and lower-cost devices have gained attention because they are more accessible and easier to integrate into routine practice [6]. However, increased availability does not automatically ensure comparable measurement performance.

For facial analysis to be clinically meaningful, scanned images must allow accurate and reproducible identification of soft tissue landmarks [7]. Even small landmark placement errors can influence linear, angular, and proportional measurements used in diagnosis and treatment planning [8]. In addition, reproducibility may vary according to landmark definition, facial region, scanner type, and examiner experience [9]. The available literature includes multiple validation and reproducibility studies, but their findings are spread across different devices, populations, and outcome measures, making direct interpretation difficult.

Therefore, this systematic review aimed to compare the ability of different 3D face scanning technologies to accurately and consistently identify facial soft tissue landmarks. The review aims to summarize their strengths, limitations, and clinical applicability, and to provide a clearer evidence-based perspective for selecting appropriate facial imaging modalities in orthodontic and dentofacial practice.

## Review

Methodology

This systematic review was conducted and reported in accordance with the PRISMA (Preferred Reporting Items for Systematic Reviews and Meta-Analyses) statement [10]. The protocol was prospectively registered in the PROSPERO database (CRD42025628750).

Review Question and Eligibility Criteria

The review question was defined as: How accurate and reproducible are facial soft tissue landmark assessments across various 3D face scanning modalities? Eligibility criteria were framed to include original studies that evaluated facial soft tissue landmark identification using one or more 3D facial scanning technologies and reported outcomes related to accuracy, reproducibility, repeatability, or reliability. Studies were eligible if they assessed modalities such as structured-light scanning, laser scanning, photogrammetry, stereophotogrammetry, smartphone-based depth scanning, or comparable 3D facial imaging systems.

Studies were excluded if they were case reports, narrative reviews, expert opinions, letters, conference abstracts without sufficient data, or methodological papers that did not report reproducibility or accuracy data relevant to facial soft tissue landmarks. Studies focused primarily on facial asymmetry method comparison, surface registration algorithms, or regional surface deviation without landmark-based outcomes as a principal component were excluded from the final synthesis. Participants of any age and gender were eligible. However, studies involving participants with major facial anomalies, syndromic craniofacial conditions, or recent facial surgery were excluded where such conditions would compromise the comparability of landmark-based reproducibility. In studies using clinical imaging populations, inclusion was accepted if relevant craniofacial exclusions were clearly applied and the outcomes directly addressed the performance of soft tissue landmark measurements.

Information Sources and Search Strategy

A comprehensive electronic literature search was performed in PubMed, Scopus, Web of Science, Semantic Scholar, and Google Scholar (first 300 sources sorted according to relevance). Each database was searched from inception to January 2026. The search strategy combined controlled vocabulary terms (where available) and free-text keywords related to 3D facial scanning, reproducibility, accuracy, and facial soft tissue landmarks. Core search concepts included terms equivalent to “3D face scanning,” “3D facial scanning,” “three-dimensional face scanning,” “reproducibility,” “repeatability,” “reliability,” “accuracy,” “facial soft tissue landmarks,” and modality-specific terms such as “structured light scanning,” “laser scanning,” “photogrammetry,” and “stereophotogrammetry.” In addition to database searching, reference lists of included studies were screened manually to identify any additional eligible records missed during the primary search. Duplicate records were identified and removed before screening.

Study Selection

Study selection was performed in two sequential stages: title/abstract screening followed by full-text screening. Two reviewers independently screened all retrieved records against the predefined eligibility criteria. At the title and abstract stage, clearly irrelevant studies were excluded. Full texts were obtained for potentially eligible records and assessed independently by both reviewers using the same criteria. Disagreements at either screening stage were resolved through discussion and consensus. When consensus was not reached, a third reviewer adjudicated the decision. Reasons for exclusion at the full-text stage were recorded systematically to ensure transparency and reproducibility of the selection process.

Data Extraction and Data Management

Data extraction was performed independently by two reviewers using a standardized extraction form designed for this review. The form was developed to capture methodological and outcome-level details necessary for a structured comparison of scanning modalities and landmark performance. Extracted study characteristics included author, year, country, study design, sample size, age, gender distribution, participant selection criteria, and scanning modality (device/system). Comparator or reference standards were also extracted in detail, including direct anthropometry, manual measurements, stereophotogrammetry reference systems, geometric references, electromagnetic digitizers, two-dimensional photographs, and cone-beam computed tomography (CBCT)-derived soft tissue models, where applicable.

For outcome extraction, the reviewers recorded the number and type of facial landmarks assessed, the outcome domains studied (accuracy, trueness, precision, reproducibility, repeatability, intra-observer reliability, inter-observer reliability), the measurement framework used, and the statistical methods reported by the authors. Accuracy-related data included, where available, mean differences, mean absolute differences, root mean square (RMS) or root mean square error (RMSE), technical error of measurement (TEM), concordance statistics, and p-values. Reproducibility-related data included intraclass correlation coefficients (ICC), Pearson correlation coefficients, kappa statistics, Bland-Altman agreement results, standard deviations of repeated landmark placement, and repeated-scan variability. Extracted data were cross-checked for consistency, and discrepancies were resolved by re-reviewing the full text and reaching consensus.

Outcome Definitions and Operational Framework

The primary outcomes of the review were accuracy and reproducibility of facial soft tissue landmark assessment using 3D facial scanning modalities. Accuracy was defined as the degree of agreement between the scan-derived landmark measurements and an external comparator or reference standard, such as direct anthropometry, validated imaging systems, or reference objects. Reproducibility was defined as the consistency of landmark identification or derived measurements across repeated observations, including intra-observer, inter-observer, and repeated-capture repeatability.

Because studies used diverse outcome terminology, related concepts such as trueness, precision, repeatability, and reliability were extracted and interpreted within a common measurement framework. Studies reporting primarily coordinate reproducibility, inter-landmark distances, angular measures, or mixed landmark-plus-surface outputs were included if the outcomes were directly relevant to soft tissue landmark assessment performance.

Risk-of-Bias Assessment

Methodological quality was assessed using the QUADAS-2 (Quality Assessment of Diagnostic Accuracy Studies-2) tool [11], adapted for measurement validation and reproducibility studies in facial imaging. Four domains were evaluated: patient selection, index test, reference standard, and flow and timing. In reproducibility-focused studies without a conventional external reference, the reference standard domain was judged based on the appropriateness and rigor of the repeat-measurement framework relative to the stated study objective. Each domain was rated for risk of bias as low, high, or unclear, and concerns regarding applicability were also judged as low, high, or unclear. Risk-of-bias assessment was performed independently by two reviewers. Domain-level disagreements were discussed and resolved by consensus. An overall study-level risk-of-bias judgment was then assigned, taking into account the pattern and severity of domain-level concerns rather than relying on a purely numerical rule.

Data Synthesis

Given the marked heterogeneity in scanner technologies, landmark sets, comparator standards, acquisition protocols, and statistical outcome measures, a meta-analysis was not considered methodologically appropriate. The review, therefore, used a structured narrative synthesis. Studies were grouped and interpreted according to imaging modality type, comparator framework, and outcome domain (accuracy and/or reproducibility). Findings were synthesized descriptively with emphasis on the direction of results, magnitude of reported errors, consistency across studies, and clinically relevant patterns such as differential reproducibility of midline versus lateral landmarks. Where appropriate, study counts were reported for key outcome categories (for example, number of studies assessing accuracy, reproducibility-only studies, and studies using direct anthropometric comparators) to provide a clear summary of the evidence distribution. Particular attention was given to differences in landmark definition, operator dependency, and regional facial variability, as these factors were repeatedly identified as sources of measurement variation.

Certainty of Evidence

The certainty of evidence was assessed using an adapted GRADE approach for diagnostic/measurement validation evidence synthesized narratively [12]. Certainty judgments were made separately for key outcome domains, including landmark accuracy/trueness, landmark reproducibility/reliability, and performance of portable or smartphone-based systems. The body of evidence was evaluated across the domains of risk of bias, inconsistency, indirectness, imprecision, and potential publication bias. Because the included studies were primarily cross-sectional measurement validation studies with heterogeneous methods and no pooled effect estimates, downgrading for inconsistency and imprecision was considered where appropriate. Final certainty ratings were expressed as high, moderate, low, or very low and were used to contextualize the strength of conclusions drawn from the narrative synthesis rather than to overstate the comparative superiority of any single modality.

Results

The electronic search yielded a total of 756 results, of which 743 were from database searches, and 13 were identified from manual reference searching through the identified articles (labelled as other sources). After de-duplication, 376 records were screened, and ultimately 18 studies were found eligible for inclusion in the present systematic review.

Study Characteristics

**Figure 1:**
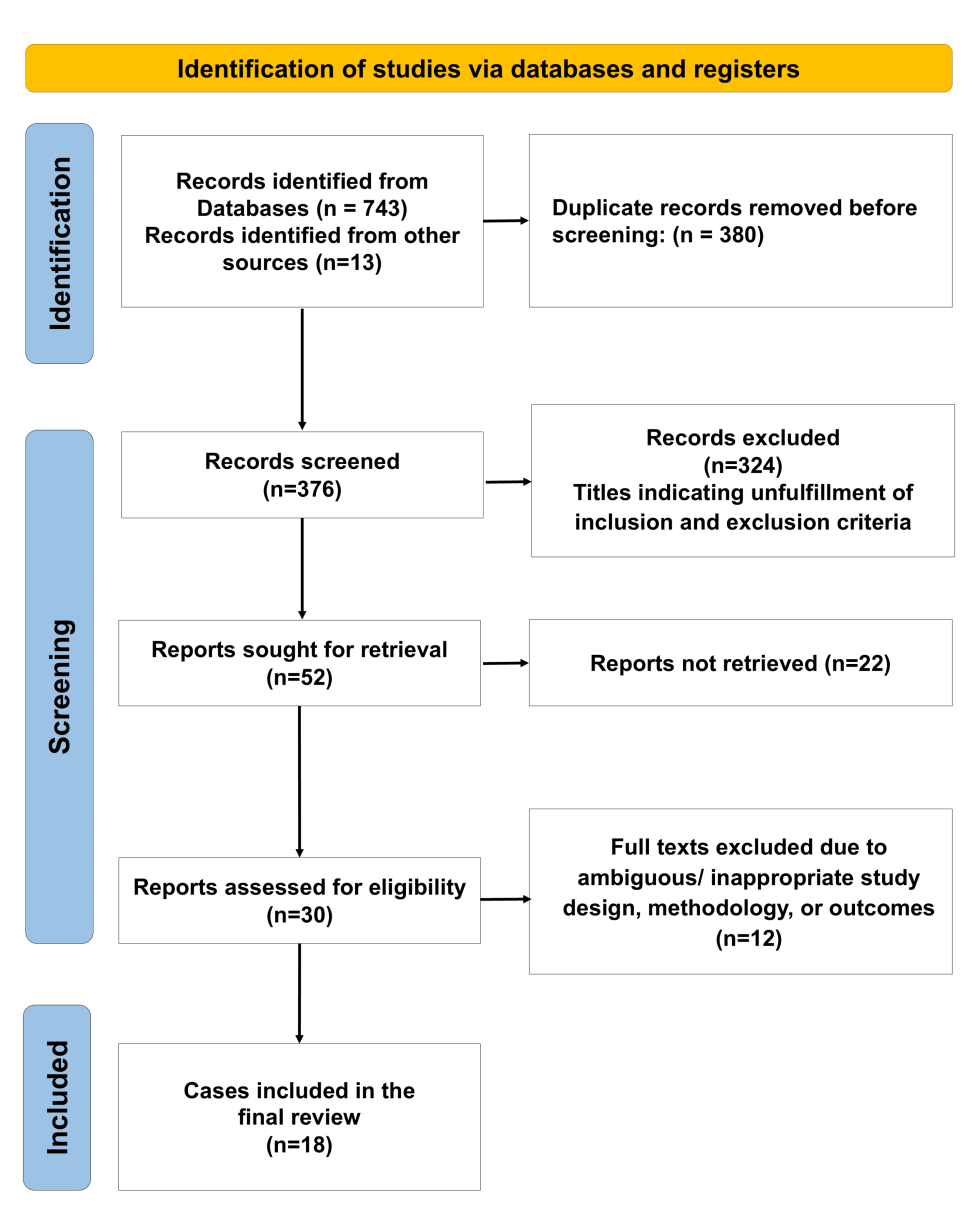
PRISMA flow diagram

A total of n=18 studies were included in the final data analysis (Figure 1), with a total pooled sample of 498 participants (n=498) [13-30]. All included studies were cross-sectional in design (n=18), conducted across various countries of North America, Europe, and Asia. The publication period extended from 2003 to 2025, showing a gradual shift from early dedicated photogrammetry and laser scanners toward stereophotogrammetry, structured-light systems, and smartphone-based facial scanning workflows. The data extracted from these studies related to their study characteristics and outcomes are summarized in Tables 1, 2.

**Table 1:** Characteristics of included studies evaluating 3D facial soft tissue landmark assessment D, two-dimensional; 3D, three-dimensional; ALSPAC, Avon Longitudinal Study of Parents and Children; CBCT, cone-beam computed tomography; M/F, male/female; NIR, near-infrared; NR, not reported; RGBD, red-green-blue-depth; y, years; N, nasion; Or, orbitale; Pg, pogonion; Po, porion; Sn, subnasale; Prn, pronasale; LS, labiale superius; Sto, stomion; LI, labiale inferius; Gn, gnathion; Al, alare; Sbal, subalare; Cph, crista philtri; Ch, cheilion.

Study (year)	Country	Study design	Sample (n)	Age	Gender (M/F)	Participant criteria	3D scanning modality (device/system)	Comparator	Landmarks assessed
Weinberg et al. (2004) [13]	USA	Cross-sectional	20	16-62 y	6/14	Healthy Caucasian individuals; no craniofacial dysmorphology	Photogrammetry (Genex 3D Camera System)	Direct anthropometry (calipers)	17 landmarks; 19 linear measurements
Gwilliam et al. (2006) [14]	UK	Cross-sectional	6 (3 adults, 3 children)	NR	NR	Healthy, non-syndromic individuals	DSP400 Facial Scanner	None (reproducibility-only study)	24 landmarks
Baik et al. (2007) [15]	Korea	Cross-sectional	60	21-27 y	30/30	Healthy Korean adults; normal occlusion; skeletal Class I; bilateral Angle Class I molar/canine relationships	Laser scanning (Vivid 900, Minolta)	None	29 landmarks
Toma et al. (2009) [16]	UK	Cross-sectional	30	Mean 15.5 y	15/15	Healthy British-Caucasian adolescents (ALSPAC cohort)	Laser scanning (Konica Minolta Vivid VI900)	None (reproducibility-only study)	21 landmarks (63 coordinates: X, Y, Z)
Menezes et al. (2009) [17]	Italy	Cross-sectional	15	22-28 y	11/4	Healthy adults; no craniofacial anomalies or prior facial surgery	Photogrammetry (PhotoModeler Pro)	3D electromagnetic digitizer (Polhemus 3Draw)	50 landmarks
Plooij et al. (2009) [18]	Netherlands & Belgium	Cross-sectional	20	23-72 y (mean 33.3)	10/10	Healthy adult Caucasian individuals; no facial deformities	Stereophotogrammetry (3dMDface)	None	49 landmarks
Menezes et al. (2010) [19]	Italy	Cross-sectional	10	20-30 y	5/5	Healthy adults; no prior craniofacial trauma/anomalies	Stereophotogrammetry (Vectra 3D)	Standard geometric object (reference distances)	16 inter-landmark distances
Aynechi et al. (2011) [20]	USA	Cross-sectional	10	Mean 32.96 ± 5.32 y	8/2	Healthy adults; no craniofacial dysmorphology/facial surgery	Stereophotogrammetry (3dMDface)	Direct anthropometry (calipers)	19 landmarks; 18 linear measurements
Othman et al. (2013) [21]	Malaysia	Cross-sectional	30	20-25 y	15/15	Healthy adults; no facial deformities	Stereophotogrammetry (VECTRA-3D dual module camera)	None (reproducibility-only study)	24 landmarks
Fink et al. (2014) [22]	Germany	Cross-sectional	32	Mean 25.5 y	19/13	Healthy adults; no craniofacial anomalies	Structured-light scanning (FaceSCAN3D Scientific Photolab 60 Hz)	None	10 landmarks
Lippold et al. (2014) [23]	Germany	Cross-sectional	15	21.8-40.6 y (mean 28.2)	5/10	Healthy adults; no craniofacial anomalies	Laser scanning (FastSCAN)	Manual anthropometric measurements	7 facial distances
Baysal et al. (2016) [24]	Turkey	Cross-sectional	34	Mean 13.1 ± 1.3 y	17/17	No prior orthodontic treatment; no craniofacial deformity/trauma	Stereophotogrammetry (3dMD Face)	None (reproducibility-only study)	19 landmarks
Fink et al. (2017) [25]	Germany	Cross-sectional	75	22-30 y (mean 23.3 ± 1.6)	35/40	Healthy adults; no craniofacial deformities	Structured-light scanning (FaceSCAN3D Scientific Photolab 60 Hz)	2D lateral photographs	5 key landmarks (N, Or, Pg, Po, Sn)
Andrews et al. (2023) [26]	Canada	Cross-sectional	29	25-50 y	6/23	Healthy adults; no craniofacial deformities	Smartphone TrueDepth NIR (iPhone 11 Pro + Bellus3D app)	3dMDface stereophotogrammetry	18 landmarks (midline + bilateral)
Gasparovic et al. (2023) [27]	Croatia	Cross-sectional	39	NR	NR	Healthy adults; no craniofacial deformities; no prior orthodontic treatment	Low-cost 3D scanner with RGBD integration	Direct physical measurements (demarcation lines)	Tragus, lateral canthus, pogonion, gonion, labial commissure
Hartmann et al. (2025) [28]	Germany	Prospective comparative (monocentric)	30	Mean 24 ± 2.3 y	15/15	Healthy adult volunteers; excluded recent craniofacial surgery/trauma and significant skeletal deformities	Smartphone 3D facial imaging (iPhone 14 Pro: TrueDepth mode and photogrammetry mode)	Vectra M5 stereophotogrammetry (reference)	15 landmarks (N, Prn, Sn, LS, Sto, LI, Gn, Al L/R, Sbal L/R, Cph L/R, Ch L/R)
Pellitteri et al. (2025) [29]	Italy	Cross-sectional (in vivo comparative)	20	Mean 26 y	11/9	Completed growth; excluded beard, facial trauma, facial esthetic surgery, skin blemishes	Hybrid structured-light scanner (EinScan H2) and stereophotogrammetry (Vectra M3)	Direct anthropometry (digital caliper) + inter-scanner comparison	6 cephalometric points (linear analysis) and 13 cephalometric landmarks (point analysis)
Megkousidis et al. (2025) [30]	USA	Cross-sectional prospective	23	18-52 y	10/13	Adults requiring CBCT; excluded craniofacial syndromes/deformities, facial trauma, facial hair	Smartphone structured-light scanning (iPhone 13 Pro Max TrueDepth + Scandy Pro app)	CBCT-derived facial soft tissue models	14 soft tissue landmarks; inter-landmark linear and angular measurements

**Table 2:** Accuracy and reproducibility outcomes of included studies 2D, two-dimensional; 3D, three-dimensional; ANOVA, analysis of variance; CBCT, cone-beam computed tomography; CCC, Lin’s concordance correlation coefficient; ICC, intraclass correlation coefficient; LoA, limits of agreement; MAD, mean absolute difference; RMSE, root mean square error; RMS, root mean square; SD, standard deviation; SE, standard error; TEM, technical error of measurement; κ, kappa coefficient; r, Pearson correlation coefficient; p, probability value; cc, cubic centimeters.

Study (year)	Outcomes assessed	Accuracy assessment (method + key result)	Accuracy statistics (reported)	Reliability/reproducibility assessment (method + key result)	Reliability statistics (reported)	Key clinical interpretation
Weinberg et al. (2004) [13]	Accuracy, reproducibility	Compared 3D photogrammetry vs direct caliper anthropometry; good agreement with minor underestimation in 14/19 variables	Significant differences in 7/19 variables (p<0.003); differences mostly <2 mm; accuracy 95.6-98.9%	Pre-labeled landmarks improved precision; low intra-/inter-observer variability	Intra-observer R=0.98; inter-observer R=0.96; MAD <1.5 mm	Genex 3D photogrammetry showed clinically acceptable accuracy and high reproducibility
Gwilliam et al. (2006) [14]	Reproducibility only	Not assessed	--	Landmark position SD in x/y/z axes; reproducibility varied by landmark and operator	Intra-operator: 12/24 landmarks SD <1 mm; inter-operator: 2/24 landmarks SD <1 mm	Reproducibility depends on landmark definition and operator familiarity; caution for poorly defined points
Baik et al. (2007) [15]	Accuracy, reproducibility	3D laser scan measurements compared with Korean anthropometric norms; overall agreement reported	Linear accuracy within ±1.2 mm	Intra-examiner repeatability across two sessions (paired comparisons) showed no significant error	p>0.01 for all; SD <1.8 mm for most landmarks	Vivid 900 laser scanning was reliable and acceptable for soft tissue landmarking in this population
Toma et al. (2009) [16]	Reproducibility only	Not assessed	--	Bland–Altman analysis for intra-/inter-examiner agreement in 3D coordinates	Intra: 51% coordinates <1 mm (38% <0.5 mm); inter: 48% <1 mm (35% <0.5 mm)	Most coordinates were reproducible within 1 mm; reproducibility should be judged in all 3 spatial planes
Menezes et al. (2009) [17]	Accuracy, reproducibility	PhotoModeler Pro vs electromagnetic digitizer; few systematic errors (2 distances, 3 angles)	MAD generally <3 mm and <3°; 5/30 comparisons significant (p<0.05)	TEM and paired testing used; digitization repeatability good, but repositioning increased error	Random error ≤1.6 mm and 3° (digitization), increasing to 5.3 mm and 5.6° with repositioning	Photogrammetry was accurate/reproducible, but subject repositioning markedly reduced reliability
Plooij et al. (2009) [18]	Reproducibility, reliability	Not assessed	--	Pearson correlation and measurement error/SE for intra-/inter-observer reliability	Intra-observer 0.97; inter-observer 0.94; mean error 0.32 mm (range 0.01-1.05 mm)	3dMD stereophotogrammetry showed high landmark reliability; midline points were more precise
Menezes et al. (2010) [19]	Accuracy, reproducibility	Vectra 3D distances compared with geometric reference object; no systematic errors	Mean difference <0.25 mm; MAD 0.13-1.19 mm; TEM <1 mm except mouth width	TEM, MAD, paired t-tests; high intra-/inter-operator reproducibility	Intra-observer MAD <1 mm in 15/16 distances; inter-observer TEM <0.7 mm; p>0.05	Vectra stereophotogrammetry provided highly accurate and reproducible soft tissue measurements
Aynechi et al. (2011) [20]	Accuracy, reproducibility	3dMDface vs caliper measurements; several statistically significant but mostly clinically small differences	Significant in 7 labeled and 6 unlabeled 3D measures (p<0.01); most errors <2 mm; larger errors in skull-base width/face width	Paired t-tests and repeated measurements; labeled landmarks improved precision	Mean absolute difference <0.5 mm for all but 2 measures; labeled 3D showed better precision (p<0.05)	3dMDface was reliable; pre-labeling improved reproducibility more than overall accuracy
Othman et al. (2013) [21]	Reproducibility only	Not assessed	--	Intra-examiner reproducibility using ICC + paired tests	ICC 0.68-0.97; no significant repeated-measurement differences (p≈0.17-0.99)	VECTRA-3D showed acceptable to high intra-examiner reproducibility; inter-examiner evaluation needed
Fink et al. (2014) [22]	Accuracy, reproducibility (precision-focused)	Intra-/interserial precision of landmark coordinates in x/y/z; all selected landmarks had high precision	Median intraserial precision 0.40 mm (range 0.05-1.01); axis medians: x 0.40, y 0.64, z 0.27 mm	Model-II ANOVA (random effects) for serial precision; interserial precision lower than intraserial	Interserial precision significantly lower; median difference 0.05 mm	Structured-light scanning (FaceSCAN3D) showed high precision with acceptable landmark reproducibility
Lippold et al. (2014) [23]	Accuracy, reproducibility	FastSCAN digital vs manual measurements using Lin’s CCC; substantial agreement for most distances	CCCs ranged from 0.752 to 0.978 (highest for outer eye corners)	Repeated scans over 3 days showed minimal variation	Repeated-scan variation <1 mm	Laser scanning was accurate and repeatable, though some landmarks were affected by image quality limitations
Baysal et al. (2016) [24]	Reproducibility only	Not assessed	--	ICCs, paired t-tests, kappa analysis for intra-/inter-examiner reproducibility	Intra-examiner ICCs 0.986-1.000 and 0.990-1.000; landmarks reproducible within 1 mm	3dMD stereophotogrammetry demonstrated very high reproducibility; z-axis agreement was strongest
Fink et al. (2017) [25]	Accuracy, reproducibility	3D structured-light scan profile analysis compared with 2D lateral photographs (Schwarz method adaptation)	Inter-method agreement κ=0.62 (moderate); intra-method 3D κ=0.84-0.94	Cohen’s kappa for intra-/inter-observer reliability; 3D showed better consistency than 2D	Intra-observer κ 0.55-0.95; inter-observer κ 0.51 (2D) vs 0.84-0.94 (3D)	3D structured-light scanning showed stronger internal consistency than 2D profile assessment
Andrews et al. (2023) [26]	Accuracy, reproducibility	iPhone TrueDepth + Bellus3D compared with 3dMDface via RMS deviation	Mean RMS 0.86 ± 0.31 mm; 97% landmarks within 2 mm; Pearson r=0.98	ICCs for intra-/inter-observer reproducibility	Intra-observer ICC 0.96 (excellent); inter-observer ICC 0.84 (good)	Smartphone-based scanning achieved clinically acceptable accuracy and good reproducibility
Gasparovic et al. (2023) [27]	Accuracy, reproducibility	Low-cost RGBD scanner vs direct physical measurements using exact distance algorithm	RMSE 2.38 mm; Pearson r=0.973; repeated scan difference <1%	ICCs for repeated measurements; repeatability varied by landmark line	ICC: tragus–pogonion 0.906-0.911 (excellent); lower/variable for gonion-based lines (including one negative ICC)	Low-cost scanning is promising, but gonion-related landmarks remain less reliable due to soft tissue variability
Hartmann et al. (2025) [28]	Accuracy, reproducibility	iPhone 14 Pro (TrueDepth and photogrammetry modes) vs Vectra M5 after surface superimposition; landmark-to-landmark and volumetric deviations assessed; photogrammetry outperformed TrueDepth overall	Overall landmark deviation: 0.8 ± 0.58 mm (photogrammetry) vs 1.1 ± 0.72 mm (TrueDepth); overall volumetric difference: 1.8 ± 2.12 cc vs 3.1 ± 2.64 cc; overall volumetric comparison p<0.001	Inter-observer agreement assessed using ICC, Bland-Altman and Wilcoxon; landmark reliability mostly good-excellent, with better volumetric consistency for photogrammetry	Landmark ICCs 0.70-0.97 (photogrammetry) and mostly 0.64-0.97 (TrueDepth); photogrammetry volumetric ICCs 0.96-0.97; pooled TrueDepth landmark inter-observer difference significant (p<0.001)	Smartphone facial scanning is clinically usable for landmarks, but photogrammetry mode showed better agreement than TrueDepth (especially for volumetric assessment)
Pellitteri et al. (2025) [29]	Accuracy, reproducibility	EinScan H2 and Vectra M3 compared with direct anthropometry and with each other (linear, point, and surface overlap analyses); no significant linear differences	Wilcoxon for linear measures p>0.05; point differences mostly within ±0.5 mm (subnasal mean 0.74 mm, borderline p=0.052); >70% area overlap within ±0.5 mm and >90% within ±1.5 mm for most areas	Repeatability/reproducibility assessed by ICC for linear and point measurements; excellent repeatability for both systems	Linear ICCs >0.90; point ICCs mostly 0.998-0.999, lowest 0.909	EinScan H2 and Vectra M3 provided comparable, accurate, and reproducible facial landmark/linear measurements
Megkousidis et al. (2025) [30]	Accuracy, reproducibility	iPhone 13 Pro Max + Scandy Pro compared with CBCT-derived facial soft tissue models using interlandmark linear/angular measurements and RMS surface analysis; no significant differences in tested measurements	Mean absolute difference: 1.43 mm (linear) and 3.16° (angular); mean RMS 1.47 mm (range 0.96-2.15); all tested linear/angular comparisons p>0.05	Intra-observer repeatability and intra-scanner precision assessed using ICC; excellent measurement reliability and good scanner precision	Mean ICCs: 0.994 (Scandy Pro) and 0.989 (CBCT); intra-scanner precision ICC 0.95 (range 0.78-1.00)	Smartphone structured-light facial scanning showed clinically acceptable accuracy/reproducibility for many applications, with lower performance in lateral facial regions

Sample sizes ranged from six to 75 participants. Age was reported in most studies (n=16), and the reported age span covered adolescents to older adults (approximately 13 to 72 years). Gender distribution was also reported in most studies (n=16), while two studies did not report gender details [14,27]. Most studies recruited healthy participants without craniofacial deformity, facial trauma, or prior facial surgery (n=17), while one study recruited adults undergoing CBCT imaging as part of routine care with relevant craniofacial exclusions applied [30].

Imaging Modalities

A broad range of 3D facial scanning technologies was evaluated. These included photogrammetry (e.g., Genex 3D, PhotoModeler Pro), laser scanning systems (e.g., Vivid 900/VI900, FastSCAN), stereophotogrammetry platforms (e.g., 3dMDface, Vectra 3D/VECTRA-3D/Vectra M3/M5), structured-light systems (e.g., FaceSCAN3D, EinScan H2), a low-cost RGBD scanner, and smartphone-based systems using TrueDepth depth capture and app-based reconstruction. This spread of modalities reflects the evolution of facial imaging from dedicated laboratory systems to more accessible clinical and consumer-grade tools.

Comparator methods were heterogeneous. Direct or manual anthropometric measurements were used in several studies (n=5) [13,20,23,27,29], including calipers and direct physical facial measurements. Other studies used instrument- or image-based reference standards (n=6) [17,19,25,26,28,30], such as an electromagnetic digitizer, geometric object reference distances, 2D lateral photographs, stereophotogrammetry reference systems, and CBCT-derived facial soft tissue models. A substantial subset focused primarily on repeatability/reproducibility without an external comparator (n=7) [14-16,18,21,22,24], particularly studies designed to evaluate landmark placement consistency.

Landmark Frameworks and Outcome Reporting

The landmark protocols varied considerably across studies. Some studies used small, focused landmark sets (e.g., five key profile landmarks), whereas others assessed broad craniofacial soft tissue landmark panels of up to 50 points [17,18,22,23,25]. Several studies reported landmark-based linear measurements, while others assessed reproducibility of x-, y-, and z-coordinate placement, inter-landmark distances, angular relationships, or derived facial distances [13,14,16,19,20,22,24,29,30]. More recent studies increasingly combined landmark-based analyses with surface or volumetric outputs, such as RMS/RMSE deviation and regional overlap analyses [26,28-30].

Outcome reporting was also methodologically diverse. Accuracy was reported using different frameworks, including direct measurement differences, MAD, TEM, CCC, RMS/RMSE, and method agreement statistics. Reproducibility/reliability was assessed using ICC, Pearson correlation, kappa statistics, Bland-Altman analysis, SD-based coordinate variability, and repeated-measurement error metrics. Because of this heterogeneity in scanners, landmark definitions, comparators, and statistical methods, a narrative synthesis was considered most appropriate.

Accuracy Outcomes

Accuracy was evaluated in 13 studies. Across the earlier literature, both photogrammetry and laser scanning systems generally demonstrated clinically acceptable agreement with reference methods. Weinberg et al. reported good agreement between 3D photogrammetry and direct anthropometry, with most differences below 2 mm despite some statistically significant differences [13]. Baik et al. similarly found laser scan measurements to be within an acceptable linear error range (±1.2 mm) [15]. Menezes et al. (2009) showed limited systematic error for photogrammetry compared with an electromagnetic digitizer, although repositioning of subjects increased measurement error and reduced consistency [17].

Stereophotogrammetry-based studies also showed favorable accuracy. Menezes et al. (2010) found very small mean differences and low MAD values using Vectra 3D against a geometric reference object, supporting high measurement precision [19]. Aynechi et al. reported statistically significant differences between 3dMDface and caliper-based measurements in some dimensions, but most errors remained clinically small (<2 mm), indicating acceptable practical performance for facial soft tissue assessment [20].

Among structured-light and later comparative studies, overall accuracy findings remained positive but varied according to comparator and outcome type. Lippold et al. demonstrated substantial agreement between FastSCAN and manual anthropometric measurements using Lin’s concordance correlation coefficients [23]. Fink et al. (2017) reported moderate agreement when a structured-light 3D method was compared with 2D profile assessment, while within-method agreement for the 3D system was stronger, suggesting better internal consistency than 2D photographic assessment [25].

Recent smartphone and hybrid-system studies also reported clinically acceptable performance in many settings. Andrews et al. found that iPhone TrueDepth imaging with Bellus3D showed a mean RMS deviation of 0.86 ± 0.31 mm compared with 3dMDface, with 97% of landmarks within 2 mm [26]. Hartmann et al. reported that both smartphone acquisition modes (TrueDepth and photogrammetry) were usable for landmark assessment against Vectra M5, but photogrammetry achieved lower overall landmark deviation and better volumetric agreement than TrueDepth [28]. Pellitteri et al. found no significant differences in linear measurements among direct anthropometry, Vectra M3, and EinScan H2, and most point differences between the two scanners were within ±0.5 mm [29]. Megkousidis et al. reported no significant differences in tested linear and angular measurements between smartphone facial scans (iPhone + Scandy Pro) and CBCT-derived soft tissue models, with a mean RMS of 1.47 mm, supporting acceptable trueness in many applications [30]. Formal between-system statistical comparisons were reported in a subset of six studies [13,17,20,28-30], and the direction and significance of these comparisons are summarized in Table 3. 

**Table 3:** Statistical significance of differences between 3D face scanning modalities and comparator methods 3D, three-dimensional; CBCT, cone-beam computed tomography; NR, not reported; RMS, root mean square.

Study (year)	Comparison (modalities/systems)	Outcome(s) compared	Statistical test(s) reported	Statistically significant difference	Key finding (direction)	Key p-value(s) reported
Weinberg et al. (2004) [13]	Photogrammetry (Genex 3D) vs direct anthropometry	Linear measurements derived from landmarks	NR	Yes (subset of variables)	Minor systematic underestimation in several variables	7/19 variables differed; p<0.003
Menezes et al. (2009) [17]	Photogrammetry (PhotoModeler Pro) vs electromagnetic digitizer (Polhemus 3Draw)	Linear distances and angles	Paired comparisons	Yes (subset of variables)	Few systematic differences between methods	5/30 measures differed; p<0.05
Aynechi et al. (2011) [20]	Stereophotogrammetry (3dMDface) vs direct anthropometry; labeled vs unlabeled 3D	Linear measurements; precision comparison	Paired t-tests; between-method comparisons	Yes (subset of measures)	Several statistically significant differences, mostly clinically small; labeled improved precision	Between-method differences: p<0.01 (subset); labeled vs unlabeled precision: p<0.05
Andrews et al. (2023) [26]	Smartphone TrueDepth (iPhone + Bellus3D) vs stereophotogrammetry (3dMDface)	Landmark deviation (RMS), correlation	Descriptive metrics only	Not reported	High agreement by RMS and correlation, but no hypothesis testing provided	NR
Hartmann et al. (2025) [28]	Smartphone TrueDepth vs smartphone photogrammetry (both vs Vectra M5 reference)	Landmark deviation; volumetric deviation	Wilcoxon signed-rank	Yes (volumetric comparison)	Photogrammetry mode showed better volumetric agreement than TrueDepth	Overall volumetric comparison p<0.001
Pellitteri et al. (2025) [29]	Structured-light (EinScan H2) vs stereophotogrammetry (Vectra M3) ± direct anthropometry	Linear and point-based measures	Wilcoxon tests	No (overall); borderline for subnasal point	No significant linear differences across methods; subnasal point difference borderline	Linear p>0.05; subnasal p=0.052
Megkousidis et al. (2025) [30]	Smartphone structured-light (iPhone + Scandy Pro) vs CBCT-derived soft-tissue model	Linear and angular inter-landmark measures	NR individually (reported as “all p>0.05”)	No	No statistically significant differences for tested measures	All p>0.05

Reproducibility and Reliability Outcomes

Reproducibility or reliability was reported in all included studies (n=18). Overall, a consistent pattern emerged across modalities: reproducibility tended to be higher for well-defined midline or sharply contoured landmarks and lower for lateral, less distinct, or soft tissue-mobile landmarks. This trend was described across multiple studies, including Gwilliam et al. [14], Toma et al. [16], Plooij et al. [18], Baysal et al. [24], and Gašparović et al. [27], and was often linked to landmark definition clarity and operator experience.

Several stereophotogrammetry studies reported high intra- and inter-observer reliability. Plooij et al. found high intra-observer and inter-observer reliability for 3dMDface with low mean measurement error, and noted better reproducibility for midline landmarks than paired landmarks [18]. Othman et al. reported acceptable to high intra-examiner reproducibility for VECTRA-3D (ICC range 0.68-0.97) [21]. Baysal et al. also reported very high reproducibility for 3dMD facial landmarks, with intra-examiner ICCs close to 1.00 and most landmarks reproducible within 1 mm [24].

Structured-light systems also showed strong repeatability in controlled settings. Fink et al. (2014) demonstrated high intra-serial precision for FaceSCAN3D, although inter-serial precision was significantly lower than intra-serial precision, indicating some variability between repeated capture sessions [22]. Fink et al. (2017) further showed better intra- and inter-observer agreement for 3D structured-light profile evaluation than for 2D photographic assessment [25].

Reproducibility remained favorable in more recent low-cost and smartphone-based systems, although with region-specific limitations. Andrews et al. reported excellent intra-observer and good inter-observer reproducibility for an iPhone-based TrueDepth workflow [26]. Hartmann et al. found mostly good-to-excellent inter-observer reliability for both smartphone modes, with photogrammetry generally performing better than TrueDepth, especially for volumetric consistency [28]. Pellitteri et al. reported excellent ICCs for both linear and point measurements for EinScan H2 and Vectra M3, supporting high repeatability of both systems [29]. Megkousidis et al. similarly reported excellent intra-observer reliability for measurements derived from both smartphone and CBCT datasets, along with good intra-scanner precision for the smartphone system [30]. In contrast, Gašparović et al. observed strong repeatability for some measurement lines (e.g., tragus-pogonion) but more variable and sometimes poor repeatability for gonion-related lines, again emphasizing that landmark/region characteristics strongly influence reproducibility [27].

Risk-of-Bias Assessment

**Figure 2:**
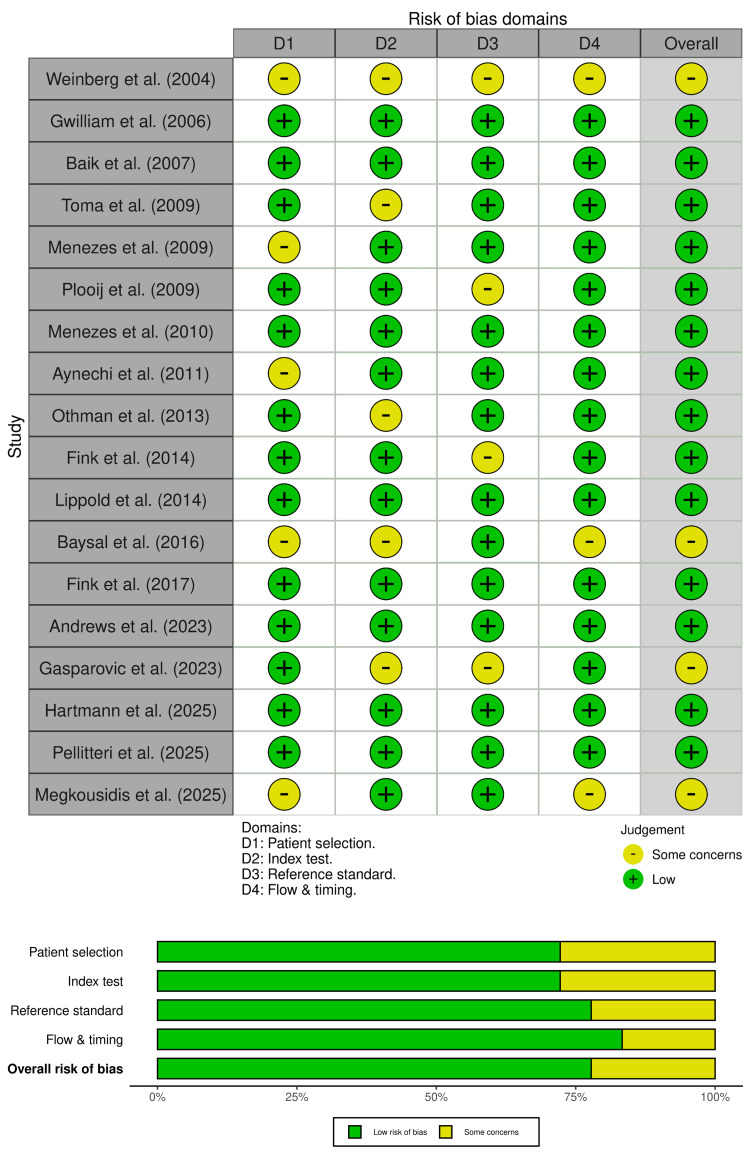
Risk of bias across the included studies using the QUADAS-2 tool [13-30].

Using QUADAS-2, most included studies were judged to have an overall low risk of bias (n=14), while the remainder (n=4) showed some concerns (Figure 2) [13,24,27,30]. Across domains, the most frequent reasons for downgrading were related to patient selection and index test conduct/reporting, with some concerns noted for patient selection in a subset of studies (n=5) [13,17,20,24,30] and for the index test domain in several studies (n=5) [13,16,21,24,27], typically reflecting limited clarity on sampling strategy, operator masking, or standardization of landmarking procedures. Reference standard concerns were less common (n=4) [13,18,22,27], generally arising when the comparator framework or reference method was not fully described or could introduce measurement variability. Flow and timing were largely low risk (n=15), with only a small number of studies showing some concerns (n=3) [13,24,30]. Overall, the risk-of-bias profile supports confidence in the direction of findings, while indicating that improved reporting and standardization would strengthen the evidence base.

Certainty of Evidence

The certainty of evidence was judged as moderate for landmark reproducibility/reliability and low for landmark accuracy/trueness, smartphone/portable system performance, and agreement with direct/manual measurements (Table 4). The main reasons for downgrading across outcomes were methodological heterogeneity (scanner types, landmark protocols, comparator standards, and statistical metrics), frequent unclear reporting in risk-of-bias domains (especially sampling and measurement procedures), and imprecision related to small sample sizes with no pooled effect estimates. Despite these limitations, the direction of findings was broadly consistent across studies, with most reports supporting clinically acceptable performance of established 3D systems and promising, but less certain, performance of newer portable/smartphone-based modalities.

**Table 4:** GRADE certainty of evidence for key outcomes

Outcome	Studies (n)/Participants (n)	Study design (body of evidence)	Risk of bias	Inconsistency	Indirectness	Imprecision	Publication bias	Overall certainty (GRADE)	Summary judgment
Accuracy/trueness of facial soft tissue landmark assessment across 3D modalities (vs reference/comparator methods)	13/378	Cross-sectional comparative/validation studies	Serious (mainly unclear reporting in sampling/blinding/thresholds)	Serious (heterogeneous scanners, landmarks, comparators, metrics)	Not serious	Serious (small study sizes; no pooled estimate)	Undetected (cannot be ruled out)	Low (⊕⊕○○)	Most studies reported clinically acceptable agreement, but certainty is limited by heterogeneity and small samples
Reproducibility/reliability of facial soft tissue landmark identification (intra-/inter-observer and/or repeated capture)	18/498	Cross-sectional reproducibility/validation studies	Serious (frequent unclear reporting of examiner masking/repeat protocols)	Not serious to serious (direction of effect broadly consistent, but metrics and landmark sets varied)	Not serious	Serious (many small studies; no pooled precision estimate)	Undetected (cannot be ruled out)	Moderate (⊕⊕⊕○)	Evidence consistently supports acceptable-to-high reproducibility, especially for well-defined landmarks
Accuracy and reproducibility of smartphone/portable/low-cost systems versus reference methods	4/121	Cross-sectional comparative studies	Not serious to serious (generally better reporting in recent studies, but not uniform)	Serious (different devices, workflows, regions, and reference standards)	Serious (mixed comparators; some outcomes combined landmark and surface/volumetric measures)	Serious (small samples per study)	Undetected (cannot be ruled out)	Low (⊕⊕○○)	Portable systems show promising performance, but certainty remains limited for broad clinical generalization
Agreement with direct/manual anthropometric or physical measurements (subset)	5/104	Cross-sectional comparative studies	Serious (older studies often incompletely reported methods)	Serious (different landmarks/distances and manual reference techniques)	Not serious	Serious (small samples)	Undetected (cannot be ruled out)	Low (⊕⊕○○)	Agreement is generally favorable, but methodological variability reduces confidence in pooled inference

Discussion

The findings of this systematic review indicate that a broad range of 3D facial scanning systems can provide a clinically acceptable assessment of facial soft tissue landmarks when used under standardized conditions. Across the included studies, reproducibility was reported more consistently than absolute accuracy, and the overall direction of evidence was favorable for established stereophotogrammetry and structured-light systems. At the same time, newer smartphone-based and low-cost systems also showed promising performance, particularly for landmark-level measurements, although their results were more variable across facial regions and outcome frameworks. This pattern is clinically relevant because it suggests that high-quality facial landmark assessment is no longer limited to large dedicated imaging setups, but the choice of device still matters depending on the intended clinical use [31].

A consistent observation across modalities was that landmark performance depends strongly on landmark anatomy. Midline and well-defined landmarks such as nasion, pronasale, and subnasale tended to show better repeatability, while lateral landmarks or points located on softer and less sharply defined contours showed greater variability [16]. This is biologically and methodologically expected. Landmarks located on stable contours are easier to identify repeatedly, whereas landmarks influenced by facial soft tissue thickness, expression, posture, or boundary ambiguity are more vulnerable to operator-related variation [31]. The same reasoning explains why some studies found stronger reproducibility in selected regions or axes and weaker results in gonion-related or lateral measurements. Therefore, scanner performance should not be interpreted as a single uniform value across the entire face.

The review also highlights that methodological differences across studies substantially influence reported outcomes. Accuracy was assessed using direct anthropometry, electromagnetic digitizers, geometric objects, stereophotogrammetry reference systems, CBCT-derived soft tissue models, and two-dimensional photographs [7,32]. These comparators are not equivalent, and each introduces its own limitations. Similarly, studies varied in whether they analyzed landmark coordinates, inter-landmark distances, angular measures, RMS/RMSE values, or mixed surface and landmark metrics. This heterogeneity explains why direct comparison between studies was difficult and why meta-analysis was not appropriate. It also means that a scanner reported as “accurate” in one framework may not show the same performance when tested against a different reference or in a different facial region.

From a clinical perspective, the evidence supports the use of validated 3D facial scanning systems for orthodontic and dentofacial soft tissue assessment, especially when repeated measurements and documentation are required. Established stereophotogrammetry and structured-light systems appear to offer strong and consistent performance for detailed facial analysis [3,7]. Smartphone-based systems may be particularly useful for screening, follow-up records, patient communication, and situations where cost or portability is important [33]. However, clinicians should remain cautious when relying on these systems for highly precise measurements in lateral facial regions or when treatment decisions depend on small landmark changes. Standardized capture conditions, operator training, and consistent landmark definitions remain essential regardless of device type [9].

This review has limitations. All included studies were cross-sectional, and many had relatively small sample sizes. Reporting quality was variable, particularly for sampling methods, blinding, and repeat-measurement protocols. The included populations were also heterogeneous in age and study setting, and the outcome metrics were not standardized. In addition, some recent studies combined landmark-based and surface-based analyses, which reflects current practice but complicates comparison. Future research should focus on larger multicenter validation studies using standardized landmark sets, uniform reporting of acquisition and landmarking protocols, and common comparator frameworks. Studies should also evaluate real-world clinical conditions, including repeated visits, operator variability, and different facial phenotypes. Such work would improve comparability across devices and help define practical thresholds for clinically meaningful measurement error.

## Conclusions

This systematic review indicates that multiple 3D facial scanning modalities can provide clinically acceptable assessments of facial soft-tissue landmarks under standardized conditions. Established stereophotogrammetry and structured-light systems showed consistently strong performance, while smartphone-based systems demonstrated promising but more variable accuracy and reproducibility across facial regions. Interpretation should remain cautious because of methodological heterogeneity and non-uniform outcome reporting across studies.

## References

[1] Karatas OH, Toy E (2014). Three-dimensional imaging techniques: A literature review. Eur J Dent.

[2] Bagheri Z, Mollabashi V, Maleki MM, Alafchi B (2025). Evaluation of facial proportions, landmarks relationships with facial and dental midlines, and smile framework. Clin Exp Dent Res.

[3] Tangthaweesuk N, Raocharernporn S (2025). The accuracy of three-dimensional facial scan obtained from three different 3d scanners. PLoS One.

[4] Aimar A, Palermo A, Innocenti B (2019). The role of 3D printing in medical applications: A state of the art. J Healthc Eng.

[5] Burlacu Vatamanu OE, Cristache CM, Drafta S, Nimigean VR (2026). Evaluation of four 3D facial scanning technologies: From photogrammetry to structured-light systems in clinical dentistry. Dent J (Basel).

[6] Gibelli D, Pucciarelli V, Cappella A, Dolci C, Sforza C (2018). Are portable stereophotogrammetric devices reliable in facial imaging? A validation study of VECTRA H1 device. J Oral Maxillofac Surg.

[7] Quinzi V, Polizzi A, Ronsivalle V (2022). Facial scanning accuracy with stereophotogrammetry and smartphone technology in children: A systematic review. Children (Basel).

[8] Zogheib T, Jacobs R, Bornstein MM, Agbaje JO, Anumendem D, Klazen Y, Politis C (2018). Comparison of 3D scanning versus 2D photography for the identification of facial soft-tissue landmarks. Open Dent J.

[9] Alves RV, Francisco H, Pinto AC, Caramês GB, Caramês J, Marques D (2026). Facial landmarks determination with different digital scanners: An in vivo study. J Clin Med.

[10] Page MJ, McKenzie JE, Bossuyt PM (2021). The PRISMA 2020 statement: An updated guideline for reporting systematic reviews. BMJ.

[11] Schueler S, Schuetz GM, Dewey M (2012). The revised QUADAS-2 tool. Ann Intern Med.

[12] Hultcrantz M, Rind D, Akl EA (2017). The GRADE Working Group clarifies the construct of certainty of evidence. J Clin Epidemiol.

[13] Weinberg SM, Scott NM, Neiswanger K, Brandon CA, Marazita ML (2004). Digital three-dimensional photogrammetry: Evaluation of anthropometric precision and accuracy using a Genex 3D camera system. Cleft Palate Craniofac J.

[14] Gwilliam JR, Cunningham SJ, Hutton T (2006). Reproducibility of soft tissue landmarks on three-dimensional facial scans. Eur J Orthod.

[15] Baik HS, Jeon JM, Lee HJ (2007). Facial soft-tissue analysis of Korean adults with normal occlusion using a 3-dimensional laser scanner. Am J Orthod Dentofacial Orthop.

[16] Toma AM, Zhurov A, Playle R, Ong E, Richmond S (2009). Reproducibility of facial soft tissue landmarks on 3D laser-scanned facial images. Orthod Craniofac Res.

[17] de Menezes M, Rosati R, Allievi C, Sforza C (2009). A photographic system for the three-dimensional study of facial morphology. Angle Orthod.

[18] Plooij JM, Swennen GR, Rangel FA (2009). Evaluation of reproducibility and reliability of 3D soft tissue analysis using 3D stereophotogrammetry. Int J Oral Maxillofac Surg.

[19] de Menezes M, Rosati R, Ferrario VF, Sforza C (2010). Accuracy and reproducibility of a 3-dimensional stereophotogrammetric imaging system. J Oral Maxillofac Surg.

[20] Aynechi N, Larson BE, Leon-Salazar V, Beiraghi S (2011). Accuracy and precision of a 3D anthropometric facial analysis with and without landmark labeling before image acquisition. Angle Orthod.

[21] Othman SA, Ahmad R, Mericant AF, Jamaludin M (2013). Reproducibility of facial soft tissue landmarks on facial images captured on a 3D camera. Aust Orthod J.

[22] Fink M, Medelnik J, Strobel K, Hirschfelder U, Hofmann E (2014). Metric precision via soft-tissue landmarks in three-dimensional structured-light scans of human faces. J Orofac Orthop.

[23] Lippold C, Liu X, Wangdo K (2014). Facial landmark localization by curvature maps and profile analysis. Head Face Med.

[24] Baysal A, Sahan AO, Ozturk MA, Uysal T (2016). Reproducibility and reliability of three-dimensional soft tissue landmark identification using three-dimensional stereophotogrammetry. Angle Orthod.

[25] Fink M, Hirschfelder U, Hirschinger V, Schmid M, Spitzl C, Detterbeck A, Hofmann E (2017). Assessment of facial soft-tissue profiles based on lateral photographs versus three-dimensional face scans. J Orofac Orthop.

[26] Andrews J, Alwafi A, Bichu YM, Pliska BT, Mostafa N, Zou B (2023). Validation of three-dimensional facial imaging captured with smartphone-based photogrammetry application in comparison to stereophotogrammetry system. Heliyon.

[27] Gašparović B, Morelato L, Lenac K, Mauša G, Zhurov A, Katić V (2023). Comparing direct measurements and three-dimensional (3D) scans for evaluating facial soft tissue. Sensors (Basel).

[28] Hartmann R, Weiherer M, Nieberle F (2025). Evaluating smartphone-based 3D imaging techniques for clinical application in oral and maxillofacial surgery: A comparative study with the vectra M5. Oral Maxillofac Surg.

[29] Pellitteri F, Calza M, Baldi G, De Maio M, Lombardo L (2025). Reproducibility and accuracy of two facial scanners: A 3D in vivo study. Appl Sci.

[30] Megkousidis K, Amm E, Motro M (2025). Evaluating accuracy of smartphone facial scanning system with cone-beam computed tomography images. Bioengineering (Basel).

[31] Al-Baker B, Ju X, Mossey P, Ayoub A (2025). The accuracy of automated facial landmarking - A comparative study between Cliniface software and patch-based Convoluted Neural Network algorithm. Eur J Orthod.

[32] Fourie Z, Damstra J, Gerrits PO, Ren Y (2011). Accuracy and repeatability of anthropometric facial measurements using cone beam computed tomography. Cleft Palate Craniofac J.

[33] Lu K, Marino NE, Russell D (2018). Use of short message service and smartphone applications in the management of surgical patients: A systematic review. Telemed J E Health.

